# Epoxy-Based Structural Self-Adhesive Tapes Modified with Acrylic Syrups Prepared via a Free Radical Photopolymerization Process

**DOI:** 10.3390/polym13020189

**Published:** 2021-01-07

**Authors:** Konrad Gziut, Agnieszka Kowalczyk, Beata Schmidt, Krzysztof Kowalczyk, Mateusz Weisbrodt

**Affiliations:** Department of Chemical Organic Technology and Polymeric Materials, Faculty of Chemical Technology and Engineering, West Pomeranian University of Technology in Szczecin, 70-322 Szczecin, Poland; kgziut@zut.edu.pl (K.G.); or beata.schmidt@zut.edu.pl (B.S.); kkowalczyk@zut.edu.pl (K.K.); or mateusz.weisbrodt@zut.edu.pl (M.W.)

**Keywords:** thermoset polymers, epoxyacrylate compositions, structural adhesives, photopolymerization, aerospace

## Abstract

New modifiers (i.e., acrylic syrups; ASs) of epoxy-resin-based thermally curable structural self-adhesive tapes (SATs) were prepared via a free radical bulk polymerization (FRBP) of n-butyl acrylate, butyl methacrylate, glycidyl methacrylate, and hydroxybutyl acrylate. In the process, two kinds of UV-photoinitiators (i.e., monoacylphosphine oxide/Omnirad TPO and bisacylphosphine oxide/Omnirad 819) and various mixing speed of the monomers mixture (200–1000 rpm) were applied. The TPO-based syrups exhibited a lower copolymers content (10–24 wt%), dynamic viscosity (<0.1 Pa·s), molecular weights (*M*_n_ and *M*_w_, and polydispersity (1.9–2.5) than these with Omnirad 819. Additionally, the higher mixing speed significantly reduced monomers conversion and viscosity of ASs as well as molecular weights of the acrylate copolymers. These parameters influenced the properties of thermally uncured (e.g., adhesion) and thermally cured SATs (shear strength of aluminum/SAT/aluminum overlap joints). Better self-adhesive features were observed for SATs-TPO (based on ASs with lower monomers conversion, *M*_n_ and *M*_w_); however, a slightly higher shear strength was noted for the thermally cured SAT-819 (ASs with higher monomers conversion, *M*_n_ and *M*_w_). An impact of polydispersity of the acrylate copolymers as well as crosslinking degree of thermally cured SATs on the mechanical strength was also revealed.

## 1. Introduction

Epoxy resins (ERs) are widely used in coatings, adhesives, fiber-reinforced composites, electrical cast insulations, and other applications [1,2,3]; however, their high crosslinking density leads to low-impact and crack-propagation resistance of the materials. Many attempts have been made to improve these features, i.e., chemical and physical modification, including the incorporation of solid polymers or inorganic micro-and nanoparticles [4,5,6,7,8,9] as well as liquid (meth) acrylate monomers into uncured ERs. One of the first reports on this subject referred to in situ UV radiation copolymerization processes of n-butyl acrylate (BA) with glycidyl methacrylate (GMA) or monoethylene glycol dimethacrylate in the presence of ER. The influence of the interaction between acrylic polymer domains and an epoxy matrix (due to their chemical reactions or IPN structure creation) on internal stress reduction was proved [10]. Copolymers of butyl acrylate (BA) and glycidyl methacrylate (GMA), prepared via an in situ polymerization process, were also tested as impact modifiers of ERs [11]. Results showed that the copolymers improve the toughness of the epoxy systems, regardless of a slight reduction of the tensile modulus and glass transition temperature. Besides, triblock copolymers, i.e., poly(styrene-*b*-butadiene-*b*-methylmethacrylate) and poly(methylmethacrylate-*b*-butylacrylate-*b*-methylmethacrylate) improve fracture toughness of the epoxy materials as well [12,13,14]. Acrylate copolymers can be incorporated into ERs in the form of hybrid nanoparticles consisting of an inorganic core (silica or aluminum oxide) and a reactive methacrylate-based shell. A UV/heat dual-curable epoxy–acrylate adhesive containing the mentioned core-shell particles exhibits outstanding water resistance [15]. Applications of epoxyacrylate copolymers (based on BA, GMA, and 2-hydroxyethyl acrylate) prepared by a conventional radical polymerization process in an organic solvent [16,17,18,19] or by bulk photopolymerization [20] are also known. In the latter case, the acrylate-type modifier was used in the form of a prepolymer also called acrylic syrup (i.e., a solution of the copolymer in the unreacted monomers). Incorporation of these acrylate-based modifiers allows the preparation of structural adhesives as self-adhesive tapes (SATs) that have relatively high adhesion to bonded substrates (before their thermal curing) and very high shear strength (after the process). This type of adhesive is commonly used in the aviation industry [21,22,23,24,25]. Preparation of the structural adhesives as thin films (based on epoxyacrylate copolymers obtained by a relatively new method, i.e., free radical bulk photopolymerization process (FRBP)) was first described in the literature by the authors of this manuscript [20].

Physicochemical properties of the prepolymers (prepared via the FRBP method) strongly depend on the process parameters such as UV dose, type and concentration of a photoinitiator, and mixing speed. This technique—as the other methods of photopolymerization—is characterized by high reaction rates and low energy costs. Moreover, it does not require elevated temperature and can be realized in the absence of organic solvents [26,27,28,29,30,31,32]. Listed advantages defined photopolymerization as a pro-ecological process, especially if cheap light-emitting diodes (LEDs) are used as a source of excitation light [33,34,35,36,37,38]. Nevertheless, the FRBP method is also subject to certain limitations. One of the main drawbacks is the formation of a gel fraction. Due to the fact that this process is carried out without solvents and the radical polymerization is a highly exo-energetic reaction, it must be conducted under appropriate conditions (such as photoinitiator or irradiation doses) to achieve a useful product. The preparation of a few different products from acrylic syrups was presented in the literature; however, the FRBP process has not yet been thoroughly explored. In detail, the influence of mixing speed on properties of acrylate (co)polymers and related adhesives has not been investigated as well. It should be noted that mixing speed was recognized as early as the 20th century as a crucial parameter of bulk photopolymerization processes. In 1969, Yemin and Hill described the influence of agitation on nonuniformly photoinitiated homopolymerization of methyl methacrylate (MMA). It should be noted that they used no photoinitiator. They also reported that the rate of MMA bulk photopolymerization increases with the increase in the speed of mixing [39]. Mendiratta et al. (1975) revealed that partial illumination of a reactor charge (styrene) (at a relatively low mixing speed) causes the formation of high molecular weight polymer chains in a shaded part of a reactor [40]. It is noteworthy that the above-mentioned papers described one-monomer-type photopolymerization reactions (homopolymerization of methyl methacrylate or styrene) based on benzoin or benzoin methyl ether as “sensitizers” and the monomer conversion value never exceeded 2%.

The main aim of this study was to investigate the influence of the mixing speed value applied during the preparation of acrylic syrups (ASs) on the selected mechanical and thermal properties of epoxy-based SATs. The free radical bulk photopolymerization process (FRBP) of various (meth)acrylates was realized using UV-LED lamps surrounding the whole reactor and resulted in the formation of ASs with different solids content and molecular weight of copolymers.

## 2. Materials and Methods

### 2.1. Materials

The following components were used for the preparation of the acrylic syrups (ASs): n-butyl acrylate (BA), butyl methacrylate (BMA), 2-hydroxyethyl acrylate (HEA) (BASF, Ludwigshafen, Germany), and glycidyl methacrylate (GMA) (Dow Europe, Horgen, Germany). Acylphosphine oxides, i.e., bis(2,4,6-trimethylbenzoyl)-phenyl phosphine oxide (Omnirad 819, IGM Resins, Waalkwijk, The Netherlands), and 2,4,6-trimethylbenzoyl-diphenyl phosphine oxide (Omnirad TPO; IGM Resins, Waalkwijk, The Netherlands) were used as type I radical photoinitiators. Thermally curable double-sided SATs were compounded using ASs, the Bisphenol A-based liquid epoxy resin with an epoxy equivalent weight of 202 g/equiv. and viscosity of 25 Pa∙s (Epidian; Ciech Sarzyna, Nowa Sarzyna, Poland), the Omnirad 127 photoinitiator (IGM Resins, Waalwijk, The Netherlands), 1,6-hexanediol diacrylate (Laromer HDDA, BASF, Ludwigshafen, Germany), the epoxy-modified acrylic resin (Laromer 9023; BASF, Ludwigshafen, Germany), the Lewis acid adduct (as a latent curing agent) (Nacure Super Catalyst A218; Worleé Chemie, Hamburg, Germany), Byk 4510 as an adhesion promoter, and Byk 378 as a surface additive (Byk-Chemie, Wesel, Germany).

### 2.2. Synthesis of Acrylic Syrups

**Figure 1:**
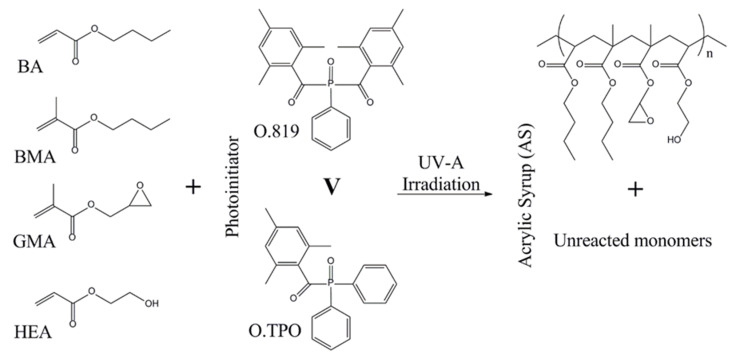
Schematic chemical structures of the tested monomers, photoinitiators, and the prepared acrylic syrups (BA: n-butyl acrylate, BMA: butyl methacrylate, GMA: glicydyl methacrylate, HEA: hydroxyethyl acrylate, O.819: Omnirad 819, O.TPO: Omnirad TPO).

ASs were prepared via the free radical bulk photopolymerization process of BA (6 moles), BMA (2 moles), GMA (1 mole), and HEA (1 mole) under UV-LED irradiation and using 0.01 mole of the photoinitiator. Chemical structures of the monomers, photoinitiators, and synthesized copolymer are shown in Figure 1. The copolymerization process was initialized at 20 °C and carried out for 20 min in a glass reactor (250 cm^3^) equipped with a mechanical stirrer and a thermocouple, and in the presence of argon as an inert gas. A mixture of the monomers (50 g) was introduced into the reactor and purged with argon for 20 min. As a UV light source, the UV-LED stripe (390 ± 5 nm; MEiSSA, Warsaw, Poland) was used. The UV irradiation density (10 mW/cm^2^) was controlled inside the reactor by means of the UV-radiometer SL2W (UV-Design, Brachttal, Germany). The composition of monomers/photoinitiator systems used for the synthesis of ASs is presented in Table 1.

### 2.3. Characterization of Acrylic Syrups

The maximum temperature (the peak temperature; *T*_max_) of the FRBP process was determined using an electronic thermometer equipped with a K-type thermocouple. Dynamic viscosity of ASs was measured at 23 °C by means of the DV-II Pro Extra viscometer (spindle #6 or #7, 50 rpm; Brookfield, New York, NY, USA). Solids content (*SC*) of the prepared syrups was determined using the MA 50/1.X2.IC.A moisture analyzer (Radwag, Radom, Poland); samples (ca. 2 mg) were heated in aluminum pans at 105 °C for 4 h. The *SC* parameter was calculated according to Equation (1),

(1) $$SC=\frac{m_{2}}{m_{1}}\cdot 100_{}\left(wt\%\right)$$

where *m*_1_ is the initial weight of a sample snf *m*_2_ is the residual weight after an evaporation process.

Gel permeation chromatography (GPC) was used for determination of molecular weights (*M*_n_, *M*_w_) and polydispersity (PDI) of the copolymers (the syrups were dried at 140 °C for 4 h before the test in order to remove unreacted monomers); the GPC apparatus contained the refractive index detector (Merck Lachrom RI L-7490), the pump (Merck Hitachi Liquid Chromatography L-7100, Abingdon, UK), the interface unit (Merck Hitachi Liquid Chromatography D-7000, Abingdon, UK), and the Shodex OHpak SB-806M MQ column connected with the Shodex OHpak SB-G pre-column (Merck Hitachi Liquid Chromatography L-7100, Abingdon, UK). The GPC tests were performed using the polystyrene standards (Fluka, Germany and Polymer Standards Service, Mainz, Germany) and tetrahydrofurane.

### 2.4. Preparation of Structural Self-Adhesive Tapes (SATs)

**Figure 2:**
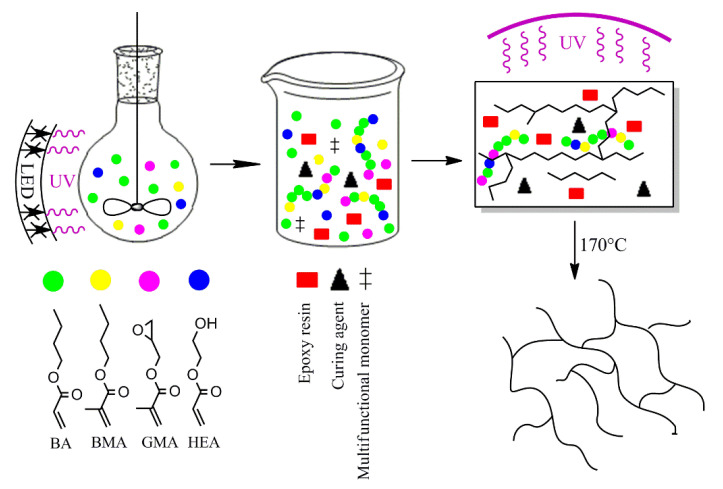
The preparation steps of the self-adhesive structural tapes (SATs).

SATs were compounded using the epoxy resin (50 wt parts), the prepared ASs (50 wt parts), the latent curing agent (1.5 wt part), the multifunctional monomers (i.e., the epoxy-modified acrylic resin; 2 wt parts), the difunctional acrylate monomer (1 wt part), the Omnirad 127 photoinitiator (3 wt parts), and the adhesion promoter (0.1 wt part). The multifunctional monomers and photoinitiators were incorporated in order to increase the crosslinking density of the UV-irradiated system. The preparation steps of SATs are graphically presented in Figure 2.

The SATs components were mixed using a high-speed mechanical mixer (T10 Basic Ultra-Turrax, IKA, Königswinter, Germany)**.** The prepared mixtures were applied onto a polyester foil (samples for self-adhesive tests) or a siliconized paper (other tests) and UV-irradiated for 30 s (8 J/cm^2^) using the medium pressure mercury lamp (UV-ABC; Hönle UV-Technology, Gräfelfing, Germany). The UV exposition was controlled with the radiometer (Dynachem 500; Dynachem Corp., Westville, IL, USA). The thickness of the prepared self-adhesive layers (i.e., the thermally uncured SATs) was ca. 120 µm.

### 2.5. Characterization of Thermally Uncured Structural Self-Adhesive Tapes (SATs)

Self-adhesive properties of the thermally uncured SATs were tested according to Association des Fabricants Européens de RubansAuto-Adhésifs (AFERA) standards, i.e., AFERA 4001 (adhesion to a steel substrate), AFERA 4015 (tack), and AFERA 4012 (cohesion). These parameters were evaluated using three samples of each adhesive tape by means of the Z010 machine (Zwick/Roell, Ulm, Germany). Generally, adhesion is defined as a force value required to remove a pressure-sensitive material from a stainless steel plate; the process is realized at the angle of 180° at a removal speed of 300 mm/min. Tack is characterized as a force value required for the separation of a stainless steel plate and an adhesive tape applied under low pressure (contact time of 0.5 s). Cohesion (i.e., static shear adhesion) describes the time needed to shear off an adhesive tape sample (under a load of 1 kg) from a defined steel surface.

Differential scanning calorimetry (DSC Q100, TA Instruments, New Castle, DE, USA) was used for determination of the glass transition temperature (*T*_g_) of SATs, enthalpy of SAT curing processes (Δ*H*), the onset temperature of the curing reactions (*T*_i_), and maximum temperature of the curing reaction (*T*_p_). Samples (ca. 10 mg) were analyzed using standard aluminum pans at the temperature from −80 to 350 °C (the heating rate of 10 °C/min). Two DSC measurements for each composition were carried out.

### 2.6. Preparation and Characterization of Aluminum Joints with Thermally Cured SATs

Aluminum–SAT–aluminum overlap joints (Al/SAT/Al) were prepared using the UV-crosslinked SATs and degreased 2024 aluminum panels (100 × 25 × 1.6 mm^3^). The joints were thermally cured at 170 °C for 60 min. Shear strength of the thermally cured Al/SAT/Al overlap joints was measured at room temperature according to the ASTMD1002-10 standard (10 samples of each system) using the Z010 machine (Zwick/Roell, Ulm, Germany). Additionally, aging tests were conducted according to the MMM-A-132B standard (shear strength of the cured overlap joints was determined after their exposition at 82 °C for 10 days). The glass transition temperature of the cured SATs was determined using the DSC method. Samples of the cured SATs (ca. 10 mg) were analyzed using standard aluminum pans at the temperature from −80 to 350 °C (the heating rate of 10 °C/min). Crosslinking degree (α) of the thermally cured SATs was calculated using the DSC data according to Equation (2) [41],

(2) $$\alpha =\left(\frac{\Delta H_{T}\;\;-\;\Delta H_{\mathrm{res}}}{\Delta H_{T}}\right)\;\left(a.u.\right)$$

where Δ*H_T_* is the total enthalpy of an SAT curing process (J/g) and Δ*H*_res_ is the enthalpy of a post-curing process of the thermally cured SAT in an Al/SAT/Al joint.

## 3. Results

### 3.1. Properties of the Acrylic Syrups

Two groups of the acrylic syrups, i.e., syrups prepared with different photoinitiator types (Omnirad TPO: monoacylphosphine oxide or Omnirad 819: bisacylphosphine oxide), were tested. These photoinitiators were used due to their relatively high absorption of the light emitted by the UV-LED lamps (the maximum absorption at 390 nm). Five syntheses were carried out for each group (10 syrups) with different mixing speeds of the reactor charge (200, 400, 600, 800, or 1000 rpm). Values of the selected physicochemical parameters of the prepared ASs (i.e., viscosity, solids content, average molecular weights, polydispersity) are presented in Table 2.

As can be seen, the mixing speed value (MS) of the reactor charge markedly affects the properties of the syrups. Generally, an increment of MS causes a reduction in the temperature peak value (*T*_max_) of the photopolymerization process (MS influences the kinetics of the process). Probably, the recorded decrement of the reaction rate (lower temperature) is caused by rapid radical extinction (reactions between two radicals resulting in polymer chain termination) and limited formation of longer/larger macroradicals. It can be confirmed by the measured viscosity of the prepared ASs, their solids content, and the molecular weights and polydispersity (PDI) of the copolymers. As can be observed, the solids content value decreases (for both product types AS-TPO and AS-819) with the increase in mixing speed value. It should be mentioned that photolysis of the Omnirad 819 photoinitiator generates four different radicals, while the TPO photoinitiator creates only two types of radicals (per one molecule) [42]. In this case, ASs prepared using Omnirad 819 are characterized by a higher viscosity, solids content, *M*_w_, and *M*_n_ values than the AS-TPO-type syrups. It was previously proved by the authors (considering NMR studies) that a solids content value directly correlates with monomers conversion in ASs [20]. The syrups based on TPO and prepared at the lowest agitation speed values (200 rpm or 400 rpm) exhibited the highest monomers conversion (24% or 21%, respectively). Under such conditions, the monomers conversion in AS-819 syrups was two-fold larger (68% and 49%). It is noteworthy that the viscosity of the AS-819-200 system was extremely high; the low MS value (200 rpm) revealed a high reactivity of the bisacylphosphine oxide photoinitiator-based system (*T*_max_ = 100 °C) and its gelling tendency. Additionally, it can be seen that the MS increment (from 600 rpm to 1000 rpm) causes only slight changes of viscosity, *SC*, molecular weights, and PDI of AS-819-type syrups. Nevertheless, it seems to be interesting that all the tested ASs reached the lowest values of these parameters exactly at MS = 600 rpm. Markedly, higher molecular weights (*M*_w_ and *M*_n_) were measured at the lower stirring speed values (200 rpm or 400 rpm) and for the Omnirad 819 photoinitiator. Moreover, these systems exhibited the highest PDI values (8.25 at 200 rpm and 3.60 at 400 rpm). For the other samples, the PDI values were lower than 2.5 (<2 for the TPO-based systems prepared at 600–1000 rpm).** It should be noted that the AS-TPO and AS-819-type syrups (MS = 600 rpm or higher) were characterized by very low viscosity (ca. 1 Pa·s or less). The significant influence of these parameters on features of the SATs is presented in further discussion.

### 3.2. Properties of the Thermally Uncured SATs

Structural self-adhesive tapes (SATs) were compounded using the Bisphenol A-type epoxy resin (ER) and the prepared acrylic syrups. After the UV-crosslinking process, their thermal properties were tested by the DSC method; the results are presented in Table 3.

**Figure 3:**
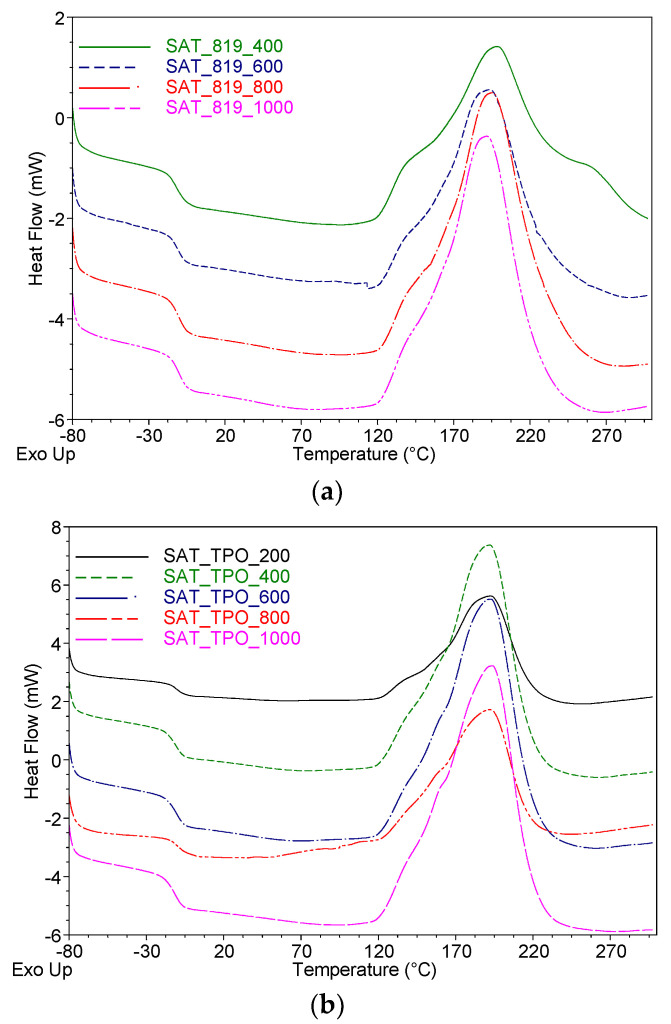
Differential scanning calorimetry (DSC) thermographs for the uncured (**a**) SATs-TPO and (**b**) SATs-819 tapes.

As can be seen, the prepared SATs exhibit similar values of glass transition temperature (*T*_g_ varies from −10 to −12 °C), and these values are not markedly affected by the physicochemical properties of the applied ASs. Nevertheless, significant differences can be observed during the thermal hardening processes of the tapes. Namely, SATs based on the ASs with a higher content of unreacted monomers (a lower *SC* value) are characterized by the wider and flatter curing peak and lower onset temperature value (*T*_i_) (Figure 3). It is especially visible for all the systems based on the AS-819 syrups exhibiting higher *SC* values (31–68%). In the case of the AS-819-200 syrup (the highest solids content), the *T*_i_ reached the relatively lowest value (107 °C), while the *T*_i_ for the AS-819-1000 system (the lowest *SC*) was 121 °C. These variations of the SATs curing processes (consisting of cationic polymerization of epoxy groups of the epoxy resin and the acrylate copolymers) can be explained by the different content of linear copolymers (i.e., flexible chains of acrylate copolymer prepared via the FRBP process) and crosslinked polyacrylate network structure (created during the UV-irradiation process of SATs). The research shows that the thermal hardening of SATs, i.e., the reaction of the epoxy groups is easier (a lower *T*_i_) in the case of the systems based on ASs with a higher solids content (a higher content of the mentioned linear copolymers than the UV-crosslinked products). Therefore, it is resulted from a lower concentration of the initial polyacrylate network in SATs. In contrast, this effect was not so markedly observed for the SAT-TPO samples (the similar *T*_i_ values: 110–114 °C) due to the relatively high content of the UV-crosslinked components (the low *SC* value for the AS-TPO syrups, i.e., <24%) and low molecular weights of the linear acrylate copolymers. For these reasons, the initial temperature of the epoxy groups polymerization in the SATs-TPO-type tapes was lower (ca. 112 °C) in relation to the SAT-819-800 and SAT-819-1000 systems (121 °C). Generally, it can be concluded that SAT-TPO materials contain more UV-crosslinked components (a denser polyacrylate network) than SATs-819. This phenomenon has a significant impact on the other parameters of SATs. Values of the temperature peak (*T*_p_) of the epoxy groups polymerization process were similar for all the SATs-TPO samples (191–193 °C) and slightly varied for SATs-819-type materials (192–198 °C). On the other hand, the values of this process enthalpy (Δ*H*) were higher for the SATs-TPO and SATs-819 systems based on the syrups with lowered *SC* parameter, i.e., the Δ*H* value increased with increasing content of the UV-crosslinked polyacrylate networks).

**Figure 4:**
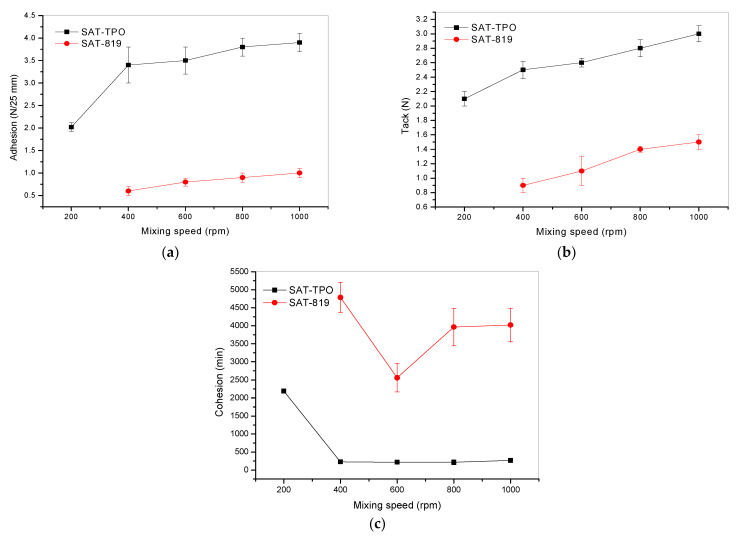
Self-adhesive features of the selected UV-crosslinked (thermally uncured) SATs: (**a**) adhesion to steel, (**b**) tack, and (**c**) cohesion at 20 °C.

Self-adhesive features of the UV-crosslinked SATs samples (i.e., adhesion to steel, tack, and cohesion) were tested before their thermal curing as well. Generally, it can be claimed that these properties depend on *SC*, the average molecular weights, and polydispersity of the acrylic syrups incorporated into SATs (while the latter parameters are affected by the mixing speed value of the reactor charge during the ASs preparation process). As can be seen, the adhesion values of SATs are relatively low (2.0–3.9 N/25 mm for SATs-TPO and 0.6–1.0 N/25 mm for SATs-819; Figure 4) and they are similar to the tack values; however, the SATs-TPO-type samples exhibited relatively better adhesion and tack. That phenomenon can be explained by the structure of SATs. The tested tapes were compounded using ca. 50 wt% of the acrylic syrup (a mixture of epoxyacrylate copolymers and unreacted monomers); the rest was the epoxy resin and the auxiliary additives. During the UV-crosslinking process, the liquid SATs compositions formed the solid double-sided pressure-sensitive films via radical photopolymerization of the acrylic compounds with double bonds. Finally, the intact epoxy resin is trapped in the acrylic matrix consisting of the epoxyacrylate copolymers and created epoxyacrylate network. In the authors’ opinion, the self-adhesive properties mainly depend on the content of linear acrylate copolymers (formed in the free radical bulk polymerization (FRBP) process) in the applied syrup and especially on their molecular weights (as it was presented in [20]); it is known that end groups of (co)polymers act as dipoles and can interact with surface groups of steel (during the adhesion or tack tests). Thus, all the SAT samples based on the AS-TPO syrups (*M*_n_ in the range of 19,840–14,120 g/mol) reached higher values of these self-adhesive features than the SAT-819-type samples (28,670–22,700 g/mol). Additionally, the higher *M*_n_ (or *M*_w_) of the selected syrup type, the better the adhesion and tack of prepared SATs. On the other hand, the SATs-819 systems exhibited much better cohesion (2559–4722 min; Figure 4c) than the SATs-TPO tapes. In this case, the mentioned parameter depends on ASs features as well. It seems that the cohesion of SATs directly depends on linear copolymer content (and its molecular weight) in the applied syrup (Table 2).

The tested SATs exhibited limited adhesion and tack; however, the values of these parameters are enough to apply them as pressure-sensitive adhesives in aluminum-aluminum overlap joints. It should be mentioned that SATs reach their final mechanical properties after their thermal curing.

### 3.3. Properties of the Thermally Cured SATs and the Al/SAT/Al Joints

**Figure 5:**
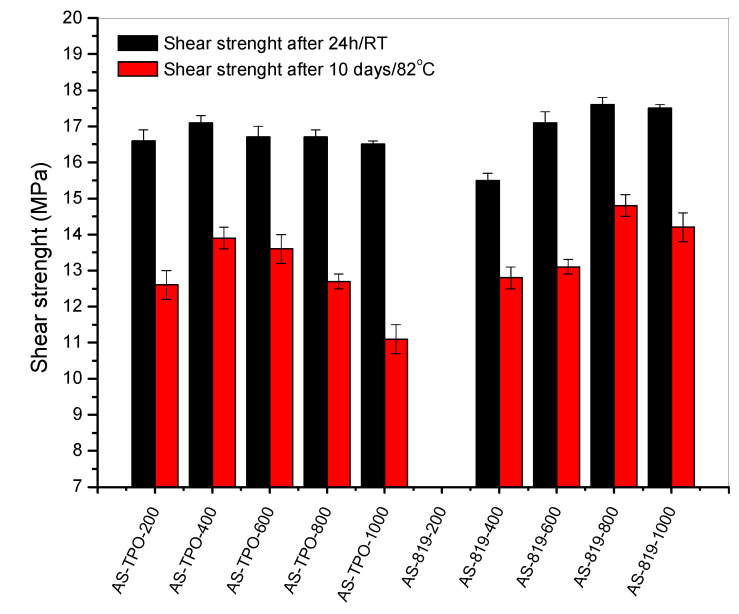
Shear strength of thermally cured aluminum–SAT–aluminum overlap joints.

The UV-crosslinked SATs were applied between aluminum panels and thermally cured at 170 °C for 60 min. Additionally, a few of the prepared Al/SAT/Al joints were thermally aged. Shear strength (τ) values for the joints (before and after thermal aging test) are presented in Figure 5.

**Figure 6:**
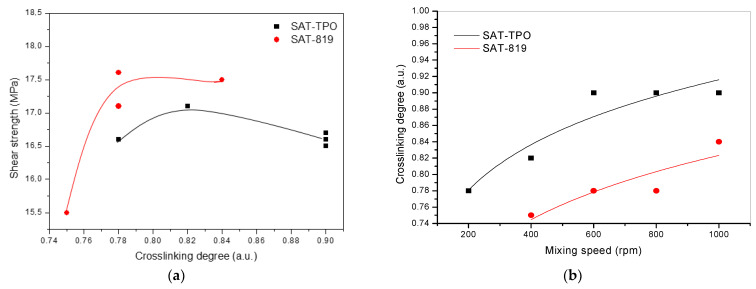
The influence of (**a**) the crosslinking degree of thermally cured SATs on overlap shear strength of Al/SAT/Al joints and (**b**) mixing speed on the crosslinking degree.

**Figure 7:**
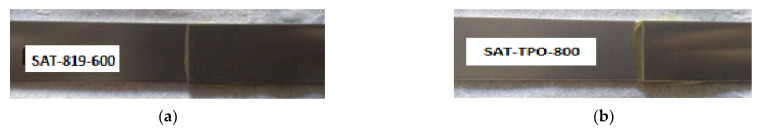
Photos of (**a**) Al/SAT-819-600/Al (slight adhesive outflow) and (**b**) Al/SAT-TPO-800/Al overlap joints (significant adhesive outflow).

As can be seen, the best shear strength (before the aging test) was recorded for the joint with SAT-819-800 (17.6 MPa), while the lowest value of this parameter was noted for SAT-819-400 (15.5 MPa). It should be noted that the latter system was based on the syrup characterized by the highest PDI value (3.6) in relation to the other samples; the thermally cured SAT-819-400 joint exhibited the lowest α value (0.75 a.u), as well. In the case of SATs-TPO-based joints, the τ values were quite similar for all the samples (16.5–17.1 MPa). It is known that the shear strength of structural adhesives depends on their crosslinking degree and an optimal range of the latter parameter value is often observed [20]. Figure 6a shows the relationship between the shear strength of Al/SAT/Al joints and the crosslinking degree of the applied SAT sample. The highest τ values for the tested joints were noted at crosslinking degree of ca. 0.78 a.u. or higher (the SATs-819-based systems) and 0.82–0.84 a.u. (the SATs-TPO-type samples). It was also found (Figure 6b) that the crosslinking degree of SATs increases with increasing mixing speed value (or with increasing content of the unreacted monomers, which finally participate in the UV-crosslinking process and increase the crosslinking density of SATs). It can be generally concluded that the higher content and molecular weights of the acrylate copolymer in the syrups (prepared via the FRBP method) positively affect the shear strength of thermally cured Al/SAT/Al joints. It is noteworthy that SATs based on syrups with lower *SC* values have a greater tendency to flow out from Al/SAT/Al joints during a thermal curing process (exemplary photos are presented in Figure 7).

Additional mechanical measurements (after the aging test) revealed a larger decrement of the shear strength of the SATs-TPO-based joints (the decrement of 24–32%) in relation to the joints with SATs-819 (reduction by 16–24%). Probably, it was affected by the increased crosslinking degree of the former SATs during the long-lasting thermal aging process. The thermally cured SATs-TPO (the unaged systems) exhibited a markedly higher value of this parameter (i.e., 0.78–0.90 a.u.) than SATs-819 (0.75–0.84 a.u.; Table 3). It was presented in references [16,20] that too high α value may deteriorate the shear strength of Al/SAT/Al overlap joints.

## 4. Conclusions

In this paper, a new preparation method (i.e., free radical bulk polymerization; FRBP) of epoxyacrylate components (acrylic syrups, ASs) for epoxy-based structural self-adhesive tapes (SATs) was presented. The influence of the applied photoinitiators (monoacylphosphine oxide/Omnirad TPO and bisacylphosphine oxide/Omnirad 819) and the mixing speed of a reactor charge (during the FRBP process) on selected features of ASs and SATs were studied.

It can be claimed that an increment of mixing speed causes a reduction of viscosity and solids content (monomers conversion) in ASs as well as molecular weights and PDI of the acrylate copolymers. Nevertheless, this relation was not significantly observed at the mixing speed higher than 600 rpm. The mentioned features influence selected parameters of SATs (prepared via UV-photopolymerization/crosslinking of mixtures consisting of ASs, an epoxy resin, multifunctional acrylate monomers, a photoinitiator, and a latent curing agent of the epoxy components). Conversion of the acrylate monomers in ASs generally represents the content of linear acrylate copolymers in SATs. It was revealed that SATs-TPO systems (based on ASs with monomers conversion lower than 24%) contain more dense polyacrylate networks because larger amounts of unreacted monomers are involved in a polymeric phase (based on multifunctional acrylate monomers) created during the UV-crosslinking process of SATs. It was also found that the cationic polymerization process (i.e., the thermal curing of UV-crosslinked SATs) occurs more easily in systems with a higher content of the linear polyacrylates (samples based on ASs with higher monomers conversion values; SATs-819) than in the presence of a dense polyacrylate network (SATs-TPO). Additionally, adhesion and tack of SATs depend on monomers conversion as well as molecular weights of the linear acrylate copolymers in the applied ASs; higher values of these self-adhesive features were observed at low monomers conversion values (<24%) and molecular weights (SATs-TPO). In the case of the thermally cured SATs (i.e., Al/SAT/Al joints), the lowest shear strength value was recorded for SAT-819-400 (15.5 MPa) based on the syrup with the highest polydispersity (3.6) while the highest value of this mechanical parameter was observed for SAT-819-800 (17.6 MPa). Thermally cured SATs-TPO systems were characterized by higher crosslinking densities (0.78–0.9 a.u.) and higher glass transition temperatures (46–51 °C) than SATs-819 (0.75–0.84 a.u. and 15–27 °C, respectively). These features increased with the increase in the mixing speed value (and with decreasing monomer conversion). Additionally, it was observed that ASs with low monomers conversion create SATs, which flowed out from the overlap Al/SAT/Al joints during their thermal hardening process. It may negatively affect the mechanical strength and aesthetics of the SAT-based overlap joints.

## Figures and Tables

**Table 1:** The composition of monomer/photoinitiator systems used for ASs preparation (at different mixing speeds).

AS Symbol	PI (0.01 mol%)	Mixing Speed (rpm)	Monomers (mol%)			
			BA	BMA	GMA	HEA
AS-TPO-200	Omnirad TPO	200	60.8	19.6	9.8	9.8
AS-819-200	Omnirad 819					
AS-TPO-400	Omnirad TPO	400				
AS-819-400	Omnirad 819					
AS-TPO-600	Omnirad TPO	600				
AS-819-600	Omnirad 819		60.8	19.6	9.8	9.8
AS-TPO-800	Omnirad TPO	800				
AS-819-800	Omnirad 819					
AS-TPO-1000	Omnirad TPO	1000				
AS-819-1000	Omnirad 819					

PI: photoinitiator; BA: n-butyl acrylate; BMA: butyl methacrylate; GMA: glycidyl methacrylate; HEA: 2-hydroxyethyl acrylate.

**Table 2:** Dynamic viscosity, solids content, average molecular weights, and polydispersity of the acrylic syrups.

AS Symbol	*T*_max_ (°C)	η (Pa∙s)	*SC* (wt%)	*M*_n_ (g/mol)	*M*_w_ (g/mol)	PDI
AS-TPO-200	40	0.2	24	19,840	48,835	2.46
AS-TPO-400	38	0.1	21	17,955	37,570	2.09
AS-TPO-600	26	<0.1	10	12,530	23,020	1.84
AS-TPO-800	30	<0.1	12	13,580	26,545	1.95
AS-TPO-1000	31	<0.1	12	14,120	26,760	1.89
AS-819-200	100	gel	68	43,515	359,085	8.25
AS-819-400	51	13	49	28,670	103,285	3.60
AS-819-600	38	0.4	31	22,380	54,985	2.46
AS-819-800	42	1.2	37	24,865	63,370	2.55
AS-819-1000	39	0.7	34	22,700	55,090	2.43

*T*_max_-process temperature peak; η—viscosity; *M*_n_—number-average molecular weight; *M*_w_—weight-average molecular weight; PDI—polydispersity index.

**Table 3:** Thermal features of the UV-crosslinked structural self-adhesive tapes (SATs), the results from the differential scanning calorimetry (DSC) tests, and the crosslinking degree of the thermally cured SATs.

SAT Symbol	*T*_g_ (°C)	*T_i_* (°C)	*T_p_* (°C)	Δ*H* (J/g)	α (a.u.)	*T*_g_ * (°C)
SAT-TPO-200	−11	114	193	211	0.78	46
SAT-TPO-400	−11	113	193	232	0.82	47
SAT-TPO-600	−11	114	192	231	0.90	51
SAT-TPO-800	−10	110	191	232	0.90	51
SAT-TPO-1000	−12	111	193	235	0.90	51
SAT-819-200	n.d.	n.d.	n.d.	n.d.	n.d.	n.d.
SAT-819-400	−12	107	198	217	0.75	15
SAT-819-600	−12	116	196	224	0.78	29
SAT-819-800	−11	121	194	230	0.78	27
SAT-819-1000	−11	121	192	238	0.84	27

*T*_g_—the glass transition temperature of uncured SATs; *T*_i_—the onset temperature of the curing reactions; *T*_p_—the maximum temperature of the curing reaction; Δ*H*—enthalpy of the SAT curing process; α—the crosslinking degree of thermally cured SATs; *T*_g_ *—the glass transition temperature for the thermally cured SATs; n.d.—no data.

## Data Availability

The data presented in this study are available on request from the corresponding author.
